# Predictors and Clinical Impact of Positive Blood Cultures in Emergency Department Patients with Suspected Infection

**DOI:** 10.3390/medicina62061104

**Published:** 2026-06-06

**Authors:** Marcello Covino, Nicola Bonadia, Luigi Celani, Davide Antonio Della Polla, Valeria Maccauro, Pierluigi Del Vecchio, Benedetta Simeoni, Antonio Gasbarrini, Rita Murri, Francesco Franceschi

**Affiliations:** 1 Emergency Department, Fondazione Policlinico Universitario A. Gemelli IRCCS, 00168 Rome, Italy; marcello.covino@policlinicogemelli.it (M.C.); nicola.bonadia@policlinicogemelli.it (N.B.);; 2 Faculty of Medicine and Surgery, Università Cattolica del Sacro Cuore, 00168 Rome, Italy; 3 Department of Infectious Diseases, Fondazione Policlinico Universitario A. Gemelli IRCCS, 00168 Rome, Italy; luigi.celani@policlinicogemelli.it; 4 Department of Internal Medicine and Gastroenterology, Fondazione Policlinico Universitario A. Gemelli IRCCS, 00168 Rome, Italy

**Keywords:** blood cultures, infection, emergency department, mortality

## Abstract

*Background and Objectives:* Blood cultures are routinely obtained in emergency department (ED) patients with suspected infection; however, their real clinical impact on patient outcomes remains debated. Although current sepsis guidelines recommend obtaining blood cultures before antimicrobial therapy, the diagnostic yield is relatively low, and it remains unclear whether early microbiological results meaningfully influence prognosis. To evaluate the predictors and clinical impact of positive blood cultures in ED patients with suspected infection and to assess whether microbiological results that modify empiric antimicrobial therapy are associated with improved survival. *Materials and Methods*: We conducted a retrospective cohort study of adult patients presenting to a tertiary-care ED with suspected infection between 2018 and 2024 who underwent blood culture sampling within the first six hours of ED stay. Blood culture results were classified as negative, positive non-actionable, or positive actionable depending on whether they led to modification of empiric antimicrobial therapy. The primary outcome was in-hospital mortality. Survival analysis was performed using Kaplan–Meier curves and multivariable Cox regression with a 72 h landmark approach to reduce immortal time bias. *Results*: The study included 13,591 patients with suspected infection who underwent blood culture testing. Blood cultures were negative in 11,475 patients, positive non-actionable in 1082 patients, and positive actionable in 1034 patients. Overall in-hospital mortality was approximately 14%. Kaplan–Meier analysis showed significant differences in survival across blood culture groups (log-rank *p* < 0.001), with lower crude survival among patients with actionable positive cultures. However, after adjustment for demographic characteristics, comorbidity burden, illness severity, and laboratory markers in multivariable Cox regression models, the presence of an actionable blood culture result was not associated with improved prognosis compared with negative or non-actionable cultures. *Conclusions*: In ED patients with suspected infection, actionable positive blood cultures were associated with higher crude mortality; however, after multivariable adjustment, this association was attenuated and did not remain statistically significant. These findings suggest that actionable blood culture results identify patients with greater illness severity and clinical complexity, while their direct patient-level survival benefit remains difficult to demonstrate in observational data. Further prospective studies are needed to clarify whether early blood culture acquisition can truly modify the clinical trajectory of patients with suspected infection.

## 1. Introduction

Sepsis and bloodstream infections remain major causes of morbidity and mortality worldwide and represent a frequent reason for emergency department (ED) evaluation among patients with suspected infection. Early recognition and timely initiation of appropriate antimicrobial therapy are key determinants of outcomes in patients with severe infections [1,2,3]. Blood cultures are traditionally considered the cornerstone of microbiological diagnosis in this setting, as they allow for identification of the causative pathogen and guide targeted antimicrobial therapy. Consequently, current international sepsis guidelines recommend obtaining blood cultures before initiating antibiotic treatment whenever feasible [2,4].

Despite these recommendations, the clinical utility of blood cultures obtained in the ED remains debated. Several studies have shown that the diagnostic yield of blood cultures in unselected ED patients with suspected infection is relatively low, with positivity rates generally ranging between 5% and 15% [5,6,7]. Moreover, a substantial proportion of positive cultures represent contaminants rather than true bloodstream infections, potentially leading to unnecessary antibiotic use, additional investigations, prolonged hospital stays, and increased healthcare costs [8,9,10]. As a result, concerns have been raised about the appropriateness of routine blood culture sampling in all patients presenting with suspected infection.

At the same time, bloodstream infections are associated with significantly increased mortality and resource utilization, particularly in patients with sepsis or septic shock [11,12]. In these cases, microbiological confirmation may have an important clinical impact by enabling pathogen-directed therapy, facilitating antimicrobial de-escalation, and identifying resistant organisms that require treatment escalation [13,14]. However, not all positive blood culture results lead to changes in clinical management. In many instances, empirical antimicrobial therapy already provides adequate coverage, and the microbiological result does not alter treatment decisions.

Recent research has therefore shifted toward evaluating clinical utility rather than the mere positivity of blood cultures. Several authors have proposed distinguishing between microbiological results that are clinically actionable, meaning they lead to a change in antimicrobial management, and those that do not influence treatment decisions [14,15,16]. This distinction is particularly relevant in the ED setting, where clinicians must balance the need for diagnostic accuracy with the risk of unnecessary testing and overtreatment.

Another important and still insufficiently explored issue concerns the impact of early blood culture collection on patient outcomes, particularly survival. While blood cultures are recommended before antibiotic administration, evidence supporting a direct survival benefit from early sampling remains limited. Most available studies have focused on bacteremia incidence, diagnostic yield, or contamination rates rather than clinical outcomes such as mortality [6,17]. Furthermore, observational studies evaluating blood culture use are often affected by indication bias, as clinicians are more likely to obtain cultures in patients with more severe clinical presentations.

Understanding the prognostic and therapeutic impact of blood cultures in real-world ED populations is therefore essential to inform diagnostic stewardship strategies. In particular, distinguishing between microbiological findings that meaningfully influence clinical management and those that do not may help identify patient subgroups most likely to benefit from early blood culture sampling.

The present study aimed to evaluate the predictors and clinical impact of positive blood cultures in a large retrospective cohort of ED patients presenting with suspected infection. Specifically, we sought to assess the association between early blood culture collection and patient survival and to determine the clinical relevance of microbiological results. By analyzing a large ED population with detailed clinical, laboratory, and outcome data, this study aims to clarify the real-world value of early blood culture testing and its role in improving outcomes among patients with suspected infection.

## 2. Methods

### 2.1. Study Design and Setting

This retrospective observational study included all consecutive adult Emergency Department presentations of Fondazione Policlinico Universitario A. Gemelli IRCCS occurring between 1 January 2018 and 31 December 2024. The study was approved by the local Ethics Committee under IRB authorization #0025817/22, issued in August 2022. Owing to the retrospective design and the use of anonymized data, the requirement for informed consent was waived.

### 2.2. Data Collection and Study Variables

Data were extracted from the ED information system and the hospital electronic medical record using standardized queries. For each eligible encounter, we collected demographic characteristics, including age and sex, comorbidity burden summarized by the Charlson Comorbidity Index [18], presenting vital signs, and the first available laboratory measurements obtained at ED presentation or during the early diagnostic workup after arrival. In particular, laboratory parameters comprised complete blood count with differential, C-reactive protein [CRP], procalcitonin [PCT], serum creatinine, blood urea nitrogen [BUN], liver enzymes, lactate dehydrogenase and coagulation markers. Hemodynamic instability was also assessed using the Shock Index (SI) [19], calculated as heart rate divided by systolic blood pressure. Organ dysfunction was summarized using the Sequential Organ Failure Assessment (SOFA) score [20], and physiological severity at ED presentation was assessed using the National Early Warning Score (NEWS) [21]. Blood culture results were retrospectively classified as actionable or non-actionable through review of the available electronic medical records and antimicrobial prescription changes [22,23,24,25]. A result was considered actionable when microbiological information was followed by a clinically relevant modification of antimicrobial management, including escalation, de-escalation, spectrum narrowing, targeted substitution, treatment discontinuation, or initiation of antimicrobial therapy. Results were considered non-actionable when microbiological findings did not modify the initial antimicrobial strategy. Because the study was retrospective, this adjudication may have been influenced by the completeness of clinical documentation. In our institution, antimicrobial therapy in patients with suspected or documented bloodstream infection is routinely discussed or reviewed by an infectious diseases team available 24/7; therefore, treatment modifications were interpreted within a specialist-led antimicrobial stewardship framework.

### 2.3. Selection of Participants

All consecutive ED visits by adult patients aged 18 years or older were screened for eligibility. Patients were included if they presented with suspected infection at ED arrival or during the initial medical assessment and had at least one blood culture obtained within the first six hours of ED stay. We excluded visits with missing key baseline variables required for the analysis, including essential laboratory parameters and organ dysfunction indices. Patients with out-of-hospital cardiac arrest without return of spontaneous circulation and those with missing outcome data were also excluded.

### 2.4. Outcomes

The primary outcome was in-hospital mortality. Secondary outcomes included the development of overt sepsis during hospitalization, defined according to the Third International Consensus Definitions for Sepsis and Septic Shock (Sepsis-3) [1].

### 2.5. Exposure

Blood culture results were classified as “actionable” when they led to a modification of empiric antimicrobial therapy and “non-actionable” when they had no impact on the initial treatment regimen. The primary exposure was the availability of an actionable microbiological result within 48 h, defined as a blood culture result leading to a clinically relevant change in management.

### 2.6. Statistical Analysis

Continuous variables are reported as medians with interquartile ranges, and categorical variables as counts and percentages. Differences across groups were assessed using the Kruskal–Wallis test for continuous variables and the chi-square test for categorical variables.

A multivariable logistic regression model was used to evaluate whether the exposure was independently associated with sepsis occurrence in the study population. The model was adjusted for variables that were statistically significant at univariate analysis and for clinically relevant covariates.

To minimize immortal time bias, the primary analysis used a 72 h landmark approach, including only patients alive and still under observation at the landmark time. A fully time-dependent Cox model was considered but was not performed because exact and systematically reliable timestamps for the availability of microbiological results and for each antimicrobial modification were not consistently available in the retrospective dataset. For this reason, the 72 h landmark analysis was selected as a pragmatic approach to reduce immortal time bias. We acknowledge that this approach cannot fully account for the exact timing of blood culture positivity or antimicrobial changes.

The analysis was adjusted for clinically relevant parameters and for variables significant at univariate analysis. Survival analyses were performed using the Kaplan–Meier method.

All analyses were conducted using SPSS version 25 (IBM Corp., Armonk, NY, USA) and custom Python 3.13 scripts. A two-sided *p*-value of less than 0.05 was considered statistically significant.

### 2.7. Ethical Statement

This study was approved by the local Ethics Committee (IRB authorization #0025817/22) and conducted in accordance with the ethical standards established in the 1964 Declaration of Helsinki and its later amendments. As all patient data were anonymized, informed consent was waived based on the study’s observational design. This retrospective observational study included all consecutive ED presentations occurring between 1 January 2018 and 31 December 2024. Ethical approval for retrospective data analysis was obtained in August 2022.

### 2.8. Artificial Intelligence Statement

During the preparation of this work, the authors used Grammarly to improve the language and readability of the manuscript. The authors reviewed and edited the content as needed and take full responsibility for the content of the publication.

## 3. Results

### 3.1. Study Population

Among 13,591 included ED visits, blood cultures were negative in 11,475 patients (84.4%), positive non-actionable in 1082 (8.0%), and positive actionable in 1034 (7.6%).

### 3.2. Baseline Characteristics

Patients with actionable cultures were older and showed a greater burden of comorbidity and physiological derangement at presentation, with higher Charlson Comorbidity Index and SOFA scores than patients with negative cultures. Baseline characteristics of the study population according to blood culture results are reported in Table 1 and Table S1. Median age was 69 years in patients with negative cultures, 72 years in those with positive non-actionable cultures, and 73 years in those with actionable cultures.

### 3.3. Crude Outcomes

Crude in-hospital mortality increased across blood culture categories, from 13.5% in patients with negative cultures to 16.2% in those with positive non-actionable cultures and 23.3% in those with positive actionable cultures.

Overall, in-hospital mortality was approximately 14.5% (1965 deaths). Patients who died during hospitalization were older and presented with higher comorbidity burden and greater organ dysfunction compared with survivors. In particular, the median age among patients who died was 76 years compared with 68 years among survivors, and the median SOFA score was 4 versus 2, respectively. Patients with in-hospital death also showed signs of more severe physiological derangement at ED presentation, including lower blood pressure, lower oxygen saturation, and higher inflammatory markers (Table S2, Table S3).

### 3.4. Survival Analysis

**Figure 1:**
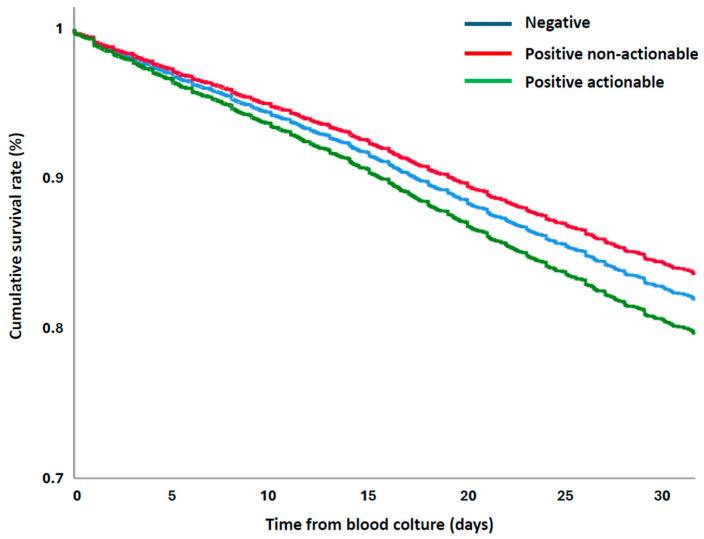
Kaplan–Meier survival curves according to blood culture results category. Cumulative survival over 30 days is shown for patients with negative blood cultures, positive non-actionable blood cultures, and positive actionable blood cultures. Numbers at risk are reported below the x-axis.

Kaplan–Meier analysis (Figure 1) demonstrated lower crude survival in the actionable group. This difference should be interpreted in light of the higher baseline severity of patients with actionable microbiological results rather than as evidence that treatment modification itself worsened prognosis.

### 3.5. Multivariable Cox Regression Analysis

In the multivariable Cox proportional hazards model adjusted for demographic variables, comorbidities, severity scores, physiological parameters, and laboratory markers, age (HR 1.021 per year, *p* < 0.001), Charlson Comorbidity Index (HR 1.10, *p* < 0.001), SOFA score (HR 1.15, *p* < 0.001), shock index (HR 1.39, *p* < 0.001), and several laboratory markers, such as procalcitonin and C-reactive protein, were independently associated with in-hospital mortality (Table 2). Compared with patients with negative blood cultures, positive non-actionable cultures were not associated with mortality (HR 0.90, 95% CI 0.76–1.06; *p* = 0.186). Positive actionable cultures showed numerically higher mortality after adjustment, but the association did not reach statistical significance (HR 1.14, 95% CI 0.99–1.32; *p* = 0.069) (Table 2). Therefore, actionable blood culture results should be interpreted as markers of higher baseline severity and clinical complexity rather than as evidence of a causal effect on mortality.

In synthesis, blood culture result group remained statistically associated with outcome after multivariable adjustment; however, residual confounding cannot be excluded.

### 3.6. Blood Culture Results

**Figure 2:**
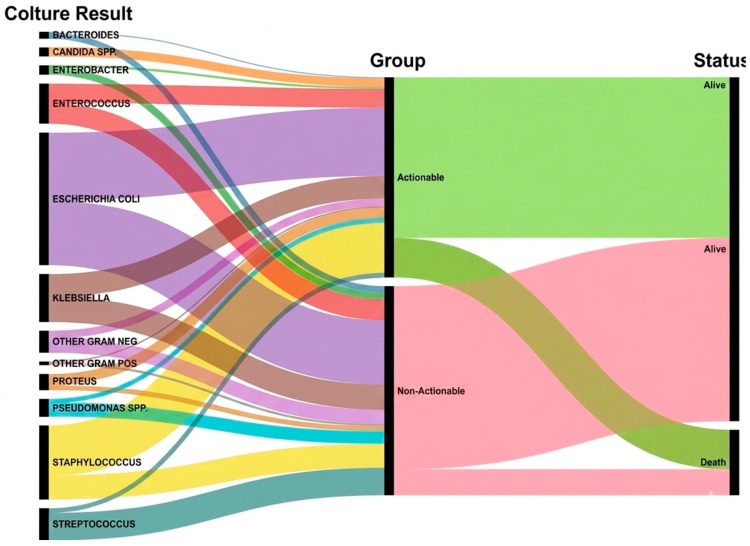
Operational classification of blood culture results. Blood culture findings were categorized as actionable when microbiological information led to a clinically relevant modification of empirical antimicrobial therapy, including escalation, de-escalation, spectrum narrowing, targeted substitution, or treatment discontinuation/initiation. Results were categorized as non-actionable when they did not alter the initial antimicrobial strategy.

The microbiological spectrum of positive blood cultures was dominated by Gram-negative bacteria (1281/2116, 60.5%), followed by Gram-positive bacteria (786/2116, 37.1%) and fungi/yeasts (49/2116, 2.3%) (Table 3). The most frequently identified individual pathogens were *Escherichia coli*, *Staphylococcus aureus complex*, *Klebsiella pneumoniae*, and *Enterococcus faecalis*. Given the low frequency of many individual species, microorganism-specific associations with outcomes were interpreted descriptively and not used for definitive prognostic inference. Indeed, as aforementioned, blood culture findings were categorized as actionable when microbiological information led to a clinically relevant modification of empirical antimicrobial therapy, including escalation, de-escalation, spectrum narrowing, targeted substitution, or treatment discontinuation/initiation (Figure 2). The blood culture result group was associated with outcome in the overall model; however, when individual categories were compared with negative cultures, actionable positive cultures showed only a non-significant trend toward higher mortality after multivariable adjustment. Residual confounding cannot be excluded.

### 3.7. Secondary Outcomes

Sepsis was recorded in 2195 patients during hospitalization. Because the retrospective dataset did not reliably distinguish sepsis already present at ED presentation from new-onset sepsis developing after admission, this secondary outcome was interpreted descriptively and should be considered exploratory. Actionable blood culture results were not associated with a clear difference in overall sepsis frequency in the available dataset.

## 4. Discussion

In this large retrospective cohort of emergency department (ED) patients with suspected infection who underwent early blood culture testing, we evaluated the clinical impact of microbiological results on patient outcomes. Several important findings emerged. First, patients with positive blood cultures, particularly those with actionable results, presented with higher comorbidity burden and greater physiological derangement at ED admission. Second, crude survival analyses demonstrated worse outcomes among patients with actionable positive cultures. However, after adjustment for demographic characteristics, comorbidities, illness severity, and laboratory parameters, this association was attenuated and did not reach conventional statistical significance. This finding suggests that actionable blood culture results primarily identify patients with more severe infection and greater clinical complexity, rather than demonstrating a direct harmful effect of treatment modification or a measurable survival benefit attributable to microbiological actionability.

Taken together, these findings suggest that although blood culture results may provide important diagnostic information, their direct impact on patient-level survival outcomes may be limited or difficult to demonstrate.

The association between bloodstream infection and increased mortality is well established. Population-based studies have consistently shown that bacteremia is linked to worse outcomes, including longer hospital stay, higher risk of organ dysfunction, and increased mortality [13,14,26,27,28]. Our findings are consistent with this body of literature, as patients with positive actionable cultures in our cohort exhibited higher baseline severity, reflected by higher SOFA scores, greater comorbidity burden, and markers of systemic inflammation. These observations support the notion that bloodstream infections frequently occur in patients with more severe underlying illness rather than being independent determinants of outcome.

However, our results also highlight an important nuance. While patients with actionable microbiological results had worse unadjusted outcomes, multivariable analyses accounting for the severity of illness and other confounders did not demonstrate a survival benefit associated with actionable culture results. In other words, the identification of a pathogen that prompted modification of empiric antimicrobial therapy did not translate into measurable improvement in patient survival. This finding raises questions about the magnitude of the patient-level benefit attributable to early microbiological diagnostics in heterogeneous ED populations.

Several recent studies have explored the concept of diagnostic stewardship in blood culture utilization [22,23,24,25]. Morgan and colleagues emphasized that blood cultures should be integrated into broader antimicrobial stewardship frameworks, as indiscriminate testing may yield limited clinical value in low-risk patients [16]. Similarly, Fabre et al. demonstrated that a substantial proportion of positive blood cultures do not lead to changes in clinical management, highlighting the need to distinguish clinically actionable results from microbiological findings that do not affect treatment decisions [14]. Our study builds on this concept by explicitly categorizing blood culture results according to their therapeutic impact and evaluating their association with survival. Therefore, this paper does not directly deal with the adequacy of empirical therapy itself.

Another explanation for the limited prognostic effect observed in our analysis may be related to the effectiveness of empiric antimicrobial therapy. In many ED patients with suspected infection, empiric regimens already provide adequate pathogen coverage. As a result, microbiological identification may confirm the appropriateness of therapy rather than altering it. Previous investigations have shown that pathogen identification frequently leads to antimicrobial de-escalation rather than escalation of therapy, reflecting adequate empiric coverage in many cases [17,29]. Consequently, the incremental survival benefit attributable to culture results may be modest in real-world clinical practice.

The timing of microbiological diagnostics may also influence their clinical impact. Rapid molecular diagnostic platforms have been shown to reduce time to targeted therapy in bloodstream infections, particularly when integrated with antimicrobial stewardship interventions [30,31]. However, the relationship between earlier pathogen identification and improved survival remains inconsistent across studies. While some trials have demonstrated improved antimicrobial optimization, effects on mortality have been less consistent, particularly in heterogeneous populations of patients with suspected infection [28].

Importantly, our findings do not suggest that blood cultures lack clinical value. Rather, they highlight that the benefit of blood culture testing may lie primarily in diagnostic clarification and antimicrobial stewardship rather than in direct survival improvement. Blood cultures remain essential for identifying resistant organisms, guiding antimicrobial de-escalation, and supporting epidemiological surveillance. In addition, microbiological data may inform infection control measures and optimize antimicrobial stewardship programs.

Our results also underscore the challenges inherent in evaluating diagnostic interventions using observational data. Patients undergoing blood culture testing often differ substantially in baseline severity compared with those who do not undergo testing. Even within tested populations, the presence of actionable microbiological results may reflect underlying illness severity rather than the causal effect of microbiological diagnostics on outcomes. Although we attempted to mitigate these biases through multivariable adjustment and landmark analysis, residual confounding cannot be excluded.

These findings have important implications for clinical practice. Given the relatively low diagnostic yield of blood cultures in ED populations and the lack of a clear survival benefit associated with actionable results in adjusted analyses, more targeted approaches to blood culture utilization may be warranted. Risk-stratification strategies incorporating clinical scores, biomarkers, and infection source may help identify patients most likely to benefit from microbiological testing.

Our findings should not be interpreted as suggesting that blood cultures are unnecessary in ED patients with suspected infection. Rather, they support a more targeted diagnostic stewardship approach, in which blood culture sampling is prioritized in patients with higher pre-test probability of bloodstream infection or greater expected therapeutic yield. This distinction is clinically relevant because blood cultures remain essential for identifying resistant pathogens, informing escalation or de-escalation of antimicrobial therapy, supporting infection-control measures, and strengthening antimicrobial stewardship programs. Accordingly, the absence of a clear patient-level survival benefit in this observational analysis should not be equated with lack of clinical utility.

Finally, our study highlights the need for further research. Prospective studies and pragmatic clinical trials are needed to determine whether early blood culture acquisition can meaningfully alter the clinical trajectory of patients with suspected infection. Future investigations should focus on identifying patient subgroups in whom microbiological diagnostics are most likely to influence treatment decisions and improve outcomes.

### Limitations

An important limitation of this study is the lack of complete and reliable timestamps for antimicrobial modifications. Although blood cultures were obtained within the first six hours of ED stay, the exact timing of microbiological result availability and subsequent treatment changes could not be systematically reconstructed. Delays in treatment modification may influence outcomes; therefore, the survival impact of actionable results could not be evaluated as a true time-dependent exposure. The 72 h landmark approach was used to reduce immortal time bias, but it remains an approximation and cannot fully replace a time-dependent Cox model. We did not have complete isolate-level susceptibility data and therefore could not formally assess the microbiological adequacy of empirical antimicrobial therapy. This represents an important limitation, because the prognostic effect of subsequent antimicrobial modifications depends strongly on whether initial empirical therapy was already active against the causative pathogen. However, in our hospital, antimicrobial management of suspected or documented bloodstream infections is supported by an infectious disease consultation team available 24/7. Thus, although formal susceptibility-based appropriateness could not be measured, treatment decisions were made within a specialist-led stewardship framework.

## 5. Conclusions

In conclusion, in this large cohort of ED patients with suspected infection, actionable positive blood cultures identified patients with greater illness severity but were not independently associated with improved survival after adjustment for confounding factors. These findings suggest that while blood cultures remain an important diagnostic tool, their direct impact on patient-level outcomes in the ED setting may be limited, and further research is needed to clarify their role in optimizing the management of suspected infection.

## Figures and Tables

**Table 1:** Study variables.

Variable	Negative	Positive Non-Actionable	Positive Actionable	*p* Value
Age, years	69 (56–79)	72 (60–80)	73 (61–81)	<0.001
Sex	6007 (52.3%)	556 (51.4%)	589 (57.0%)	0.012
Charlson comorbidity index	4 (2–6)	4 (3–6)	5 (3–7)	<0.001
SOFA score	2 (1–4)	3 (1–5)	3 (2–5)	<0.001
Ed presentation				
Symptoms within 24 h	50.5% (44.1%)	570 (52.7%)	576 (55.7%)	<0.001
Triage category				
Emergency	1152 (10.0%)	140 (12.9%)	144 (13.9%)	<0.001
Urgency	3865 (33.7%)	397 (36.7%)	403 (39.0%)	
Minor urgency	6458 (56.3%)	545 (50.4%)	487 (47.1%)	
Fever	5986 (52.2%)	554 (51.2%)	530 (51.3%)	0.730
Dyspnea	3164 (27.6%)	303 (28.0%)	269 (26.0%)	0.519
Chest pain	524 (4.6%)	62 (5.7%)	49 (4.7%)	0.221
Abdominal pain	2357 (20.5%)	210 (19.4%)	211 (20.4%)	0.677
Vomiting	1348 (11.7%)	134 (12.4%)	121 (11.7%)	0.821
Diarrhea	719 (6.3%)	63 (5.8%)	51 (4.9%)	0.210
Syncope	507 (4.4%)	53 (4.9%)	45 (4.4%)	0.755
Malaise/asthenia	1494 (13.0%)	138 (12.8%)	131 (12.7%)	0.927
Trauma	758 (6.6%)	67 (6.2%)	60 (5.8%)	0.548
Vital signs				
Heart rate, bpm	94 (82–107)	96 (83–110)	96 (83–110)	0.001
Respiratory rate, breaths/min	30 (21–39)	30 (21–39)	30 (21–39)	0.735
Glasgow Coma Scale	15 (15–15)	15 (15–15)	15 (15–15)	0.021
Systolic blood pressure, mmHg	125 (110–140)	120 (100–139)	118 (100–138)	<0.001
Diastolic blood pressure, mmHg	75 (65–84)	70 (60–80)	70 (60–80)	<0.001
Oxygen saturation, %	95 (92–97)	96 (93–98)	95 (92–97)	0.005
Temperature, C	36.5 (36.0–38.0)	37.3 (36.0–38.0)	37.1 (36.0–38.0)	<0.001
Laboratory Values				
Hemoglobin, g/dL	11.6 (10.0–13.3)	11.4 (9.8–13.0)	11.0 (9.6–12.5)	<0.001
White blood cell count	11.1 (7.5–15.9)	11.5 (7.5–17.1)	12.4 (8.3–17.7)	<0.001
Platelet count	235 (161–334)	191 (129–276)	199.5 (132.8–289.3)	<0.001
Prothrombin time	11.9 (11.2–13.3)	12.4 (11.5–14)	12.4 (11.5–14.1)	<0.001
INR	1.1 (1.03–1.23)	1.17 (1.08–1.32)	1.17 (1.08–1.34)	<0.001
Blood urea nitrogen	19 (13–31)	26 (17–39)	28 (18–43)	<0.001
Creatinine	0.99 (0.72–1.58)	1.23 (0.83–1.99)	1.29 (0.88–2.15)	<0.001
AST	21 (12–40)	25 (14–50)	21 (12–44.2)	<0.001
Total bilirubin	0.7 (0.5–1.2)	0.9 (0.6–1.8)	0.8 (0.5–1.5)	<0.001
Sodium	135 (132–138)	136 (132–139)	135 (132–139)	0.162
Potassium	4.0 (3.6–4.5)	4.0 (3.6–4.6)	4.0 (3.6–4.7)	0.001
C-reactive protein	114.2 (45–189)	145.4 (72.6–224.3)	147.1 (71–229.6)	<0.001
Procalcitonin	0.41 (0.13–2.24)	3.4 (0.47–19.2)	2.5 (0.39–21.0)	<0.001
Lactate dehydrogenase	270 (203–395)	281 (210–403)	275.50 (204–398)	0.079
Fibrinogen	569 (423–751)	579 (449–751)	584 (440–754.25)	0.058
Outcomes				
ICU admission	1575 (13.7%)	145 (13.4%)	144 (13.9%)	0.937
Sepsis	1853 (16.1%)	168 (15.5%)	174 (16.8%)	0.719
Septic shock	235 (2.0%)	28 (2.6%)	27 (2.6%)	0.272
Death	1549 (13.5%)	175 (16.2%)	241 (23.3%)	<0.001

**Table 2:** Cox regression results. BC, blood culture; CI, confidence interval; HR, hazard ratio; SOFA, Sequential Organ Failure Assessment.

Variable	Hazard Ratio (HR)	95% CI	*p*-Value
Blood culture result group			0.049
Positive non-actionable BC vs. negative BC	0.90	0.76–1.06	0.186
Positive actionable BC vs. negative BC	1.14	0.99–1.32	0.069
Sex	0.86	0.78–0.94	0.001
Symptoms within 24 h	1.04	0.95–1.15	0.380
Triage category			<0.001
Triage category level 1	0.78	0.69–0.88	<0.001
Triage category level 2	0.54	0.47–0.62	<0.001
Age, per year	1.02	1.02–1.03	<0.001
Charlson Comorbidity Index	1.10	1.08–1.12	<0.001
SOFA score	1.15	1.12–1.18	<0.001
Shock Index	1.39	1.18–1.63	<0.001
Oxygen saturation	0.99	0.99–1.00	0.108
Temperature	0.88	0.85–0.92	<0.001
Hemoglobin	0.98	0.96–1.00	0.032
White blood cell count	1.00	1.00–1.01	0.199
Neutrophil-to-lymphocyte ratio	1.01	1.00–1.01	<0.001
Blood urea nitrogen	1.01	1.01–1.01	<0.001
Creatinine	0.89	0.86–0.93	<0.001
C-reactive protein	1.001	1.001–1.002	<0.001
Procalcitonin	0.99	0.99–1.00	<0.001

**Table 3:** Distribution of identified microorganisms, grouped by major microbiological category and stratified by sepsis status. Values are reported as *n* (% within column).

Microorganism Group	No Sepsis (*n* = 11,396)	Sepsis (*n* = 2195)	Total (*n* = 13,591)
Negative cultures	9622 (84.4)	1853 (84.4)	11475 (84.4)
All positive isolates	1774 (15.6)	342 (15.6)	2116 (15.6)
Gram-negative bacteria	1072 (9.4)	209 (9.5)	1281 (9.4)
Gram-positive bacteria	656 (5.8)	130 (5.9)	786 (5.8)
Fungi/yeasts	46 (0.4)	3 (0.1)	49 (0.4)

Abbreviations: Percentages are calculated within each column. Gram-negative bacteria included *Enterobacterales,* non-fermenting Gram-negative bacilli, anaerobic Gram-negative bacteria, and other Gram-negative organisms. Gram-positive bacteria included *Staphylococcus* spp., *Enterococcus* spp., *Streptococcus* spp., and other Gram-positive organisms. Fungi/yeasts included *Candida* spp. and other fungal isolates. The total number of positive isolates was fully consistent with the overall blood culture classification reported in the manuscript.

## Data Availability

The data presented in this study are available on request from the corresponding author due to privacy and ethical restrictions.

## Supplementary Materials

The following supporting information can be downloaded at: https://www.mdpi.com/article/10.3390/medicina62061104/s1, Table S1: Categorical variables according to blood culture result group; Table S2: Continuous variables according to death status; Table S3: Categorical variables according to death status.

## Abbreviations

The following abbreviations are used in this manuscript:

BC	Blood culture
BSI	Bloodstream infection
ED	Emergency department
NEWS	National Early Warning Score
SOFA	Sequential Organ Failure Assessment
ICU	Intensive Care Unit
IRCSS	Istituto di Ricovero e Cura a Carattere Scientifico.
