# Supplementary material for: Predictors and Clinical Impact of Positive Blood Cultures in Emergency Department Patients with Suspected Infection
Source: Medicina (Kaunas). 2026 Jun 6;62(6):1104. doi: 10.3390/medicina62061104 (PMC13303041; doi:10.3390/medicina62061104)
Supplement: Supplementary file 1 [file medicina-62-01104-s001.zip › medicina-4252833 Supplementary Materials.pdf]

Supplementary Materials

Table S1. Categorical variables according to blood culture result group

Variable	Negative	Positive non-actionable	Positive actionable	p value
**Blood culture results**				
Any positive blood culture	0 (0.00%)	1082 (100.00%)	1034 (100.00%)	<0.001
Positive fungal blood culture	0 (0.00%)	0 (0.00%)	48 (4.60%)	<0.001
**ED presentation**				
Symptoms within 24 h	5055 (44.10%)	570 (52.70%)	576 (55.70%)	<0.001
**Triage category**				
Emergency	1152 (10.00%)	140 (12.90%)	144 (13.90%)	<0.001
Urgency	3865 (33.70%)	397 (36.70%)	403 (39.00%)	
Minor urgency	6458 (56.30%)	545 (50.40%)	487 (47.10%)	
Fever	5986 (52.20%)	554 (51.20%)	530 (51.30%)	0.73
Dyspnea	3164 (27.60%)	303 (28.00%)	269 (26.00%)	0.52
Chest pain	524 (4.60%)	62 (5.70%)	49 (4.70%)	0.22
Abdominal pain	2357 (20.50%)	210 (19.40%)	211 (20.40%)	0.68
Vomiting	1348 (11.70%)	134 (12.40%)	121 (11.70%)	0.82
Diarrhea	719 (6.30%)	63 (5.80%)	51 (4.90%)	0.21
Syncope	507 (4.40%)	53 (4.90%)	45 (4.40%)	0.76
Malaise/asthenia	1494 (13.00%)	138 (12.80%)	131 (12.70%)	0.93
Trauma	758 (6.60%)	67 (6.20%)	60 (5.80%)	0.55

Variable	Negative	Positive non-actionable	Positive actionable	p value
Confusion	588 (5.10%)	52 (4.80%)	62 (6.00%)	0.41
Edema	322 (2.80%)	36 (3.30%)	40 (3.90%)	0.11
Oligo-anuria	216 (1.90%)	26 (2.40%)	15 (1.50%)	0.27
**Infective focus**				
Pulmonary site	3826 (33.30%)	357 (33.00%)	339 (32.80%)	0.92
Abdominal site	2747 (23.90%)	240 (22.20%)	246 (23.80%)	0.43
Renal site	1696 (14.80%)	150 (13.90%)	160 (15.50%)	0.57
Cutaneous site	384 (3.30%)	28 (2.60%)	36 (3.50%)	0.39
Other systemic site	2184 (19.00%)	214 (19.80%)	207 (20.00%)	0.64
Non-confirmed infection	11475 (100.00%)	1082 (100.00%)	1034 (100.00%)	NR
**Number of infectious sites**				
0	2581 (22.50%)	246 (22.70%)	232 (22.40%)	0.56
1	7153 (62.30%)	699 (64.60%)	657 (63.50%)	
2	1554 (13.50%)	123 (11.40%)	129 (12.50%)	
3	175 (1.50%)	12 (1.10%)	15 (1.50%)	
4	12 (0.10%)	2 (0.20%)	1 (0.10%)	
Unconfirmed infective focus	2581 (22.50%)	246 (22.70%)	232 (22.40%)	0.98
**Comorbidities**				
Active malignancy	2436 (21.20%)	214 (19.80%)	232 (22.40%)	0.32
Atrial fibrillation	1236 (14.10%)	87 (8.00%)	115 (11.10%)	<0.001
History of MI/CAD	974 (8.50%)	125 (11.60%)	119 (11.50%)	<0.001
Heart failure	1240 (10.80%)	77 (7.10%)	68 (6.60%)	<0.001
Peripheral	477 (4.20%)	65 (6.00%)	67 (6.50%)	<0.001

Variable	Negative	Positive non-actionable	Positive actionable	p value
vascular disease				
Previous stroke/TIA	824 (7.20%)	47 (4.30%)	69 (6.70%)	0.002
Dementia	830 (7.20%)	72 (6.70%)	98 (9.50%)	0.02
COPD	843 (7.30%)	78 (7.20%)	116 (11.20%)	<0.001
Connective tissue disease	321 (2.80%)	28 (2.60%)	21 (2.00%)	0.34
Liver disease	799 (7.00%)	48 (4.40%)	48 (4.60%)	<0.001
Cirrhosis	802 (7.00%)	68 (6.30%)	52 (5.00%)	0.04
Diabetes	1706 (14.90%)	180 (16.60%)	183 (17.70%)	0.02
Complicated diabetes	399 (3.50%)	35 (3.20%)	46 (4.40%)	0.23
Hemiplegia	106 (0.90%)	5 (0.50%)	18 (1.70%)	0.01
Renal insufficiency	1547 (13.50%)	207 (19.10%)	243 (23.50%)	<0.001
Malignancy	2695 (23.50%)	275 (25.40%)	261 (25.20%)	0.19
Metastatic disease	872 (7.60%)	80 (7.40%)	61 (5.90%)	0.14
Lymphoma/leukemia	549 (4.80%)	47 (4.30%)	69 (6.70%)	0.02
Dialysis	286 (2.50%)	24 (2.20%)	15 (1.50%)	0.10
HIV infection	168 (1.50%)	9 (0.80%)	6 (0.60%)	0.02
**Outcomes**				
ICU admission	1575 (13.70%)	145 (13.40%)	144 (13.90%)	0.94
Sepsis	1853 (16.10%)	168 (15.50%)	174 (16.80%)	0.72
Septic shock	235 (2.00%)	28 (2.60%)	27 (2.60%)	0.27
Death	1549 (13.50%)	175 (16.20%)	241 (23.30%)	<0.001

Table S2. Continuous variables according to death status

Variable	No death	Death	p value
**Demographics and severity**			
Age, years	68.00 (55.00-78.00)	76.00 (66.00-84.00)	<0.001
Charlson comorbidity index	4.00 (2.00-6.00)	5.00 (4.00-7.00)	<0.001
SOFA score	2.00 (1.00-4.00)	4.00 (2.00-5.00)	<0.001
ED stay, h	38.53 (21.20-68.33)	32.20 (20.10-65.90)	0.003
Length of stay, days	12.00 (8.00-19.00)	13.00 (7.00-22.52)	0.05
Resistance among tested isolates, %	0.10 (0.00-0.33)	0.20 (0.00-0.39)	<0.001
Shock index	0.75 (0.63-0.91)	0.80 (0.65-0.99)	<0.001
**Vital signs**			
Heart rate, bpm	94.00 (82.00-108.00)	95.00 (81.00-110.00)	0.18
Respiratory rate, breaths/min	30.00 (21.00-39.00)	28.00 (20.00-37.00)	<0.001
Glasgow Coma Scale	15.00 (15.00-15.00)	15.00 (15.00-15.00)	<0.001
Systolic blood pressure, mmHg	125.00 (110.00-141.00)	119.00 (100.00-135.00)	<0.001
Diastolic blood pressure, mmHg	75.00 (65.00-84.00)	70.00 (60.00-80.00)	<0.001
Mean arterial pressure, mmHg	91.70 (81.00-102.30)	86.00 (75.00-97.70)	<0.001
Oxygen saturation, %	96.00 (92.00-98.00)	94.00 (90.00-96.00)	<0.001
Temperature, C	36.80 (36.00-38.00)	36.20 (36.00-38.00)	<0.001
PaO2, mmHg	145.50 (102.73-148.50)	139.96 (80.40-148.50)	<0.001
**Laboratory values**			

Variable	No death	Death	p value
Hemoglobin, g/dL	11.70 (10.10-13.20)	10.90 (9.40-12.60)	<0.001
White blood cell count	11.08 (7.53-15.82)	12.22 (7.91-18.29)	<0.001
Neutrophil-to-lymphocyte ratio	8.20 (4.51-14.77)	11.62 (6.29-20.05)	<0.001
Red cell distribution width, %	15.20 (14.00-16.80)	16.30 (14.90-18.10)	<0.001
Platelet count	232.00 (160.00-328.00)	214.00 (130.00-322.00)	<0.001
Prothrombin time	11.90 (11.20-13.10)	12.70 (11.60-14.70)	<0.001
INR	1.10 (1.03-1.22)	1.18 (1.08-1.38)	<0.001
Blood urea nitrogen	19.00 (13.00-29.00)	30.00 (19.00-48.00)	
Creatinine	0.99 (0.73-1.55)	1.31 (0.81-2.25)	
AST	21.00 (12.00-40.00)	23.00 (12.00-45.00)	
Total bilirubin	0.70 (0.50-1.20)	0.70 (0.50-1.30)	
Sodium	135.00 (132.00-138.00)	135.00 (130.00-139.00)	
Potassium	4.00 (3.60-4.40)	4.20 (3.70-4.90)	
C-reactive protein	115.00 (46.10-190.00)	141.90 (66.70-209.80)	
Procalcitonin	0.45 (0.13-2.90)	1.04 (0.28-5.66)	
Lactate dehydrogenase	260.00 (199.00-373.00)	345.00 (249.00-539.00)	
Activated partial thromboplastin time	30.60 (26.50-35.63)	31.60 (27.10-38.40)	
International normalized ratio	1.09 (1.03-1.20)	1.17 (1.07-1.34)	
Fibrinogen	577.50 (433.75-756.00)	538.00 (386.00-718.00)	

Table S3. Categorical variables according to death

status

Variable	No death	Death	p value
**Microbiology**			
Actionable blood culture	793 (6.80%)	195 (12.00%)	<0.001
**ED presentation**			
Symptoms within 24 h	5405 (46.50%)	658 (40.30%)	<0.001
**Triage category**			
Emergency	950 (8.20%)	717 (44.00%)	<0.01
Urgency	3805 (32.70%)	530 (32.50%)	
Minor urgency	6871 (59.10%)	383 (23.50%)	
Fever	6041 (52.00%)	843 (51.70%)	0.84
Dyspnea	3195 (27.50%)	447 (27.40%)	0.95
Chest pain	544 (4.70%)	68 (4.20%)	0.36
Abdominal pain	2408 (20.70%)	322 (19.70%)	0.36
Vomiting	1364 (11.70%)	201 (12.30%)	0.49
Diarrhea	709 (6.10%)	114 (7.00%)	0.16
Syncope	509 (4.40%)	77 (4.70%)	0.53
Malaise/asthenia	1515 (13.00%)	198 (12.10%)	0.32
Trauma	761 (6.50%)	108 (6.60%)	0.91
Confusion	590 (5.10%)	85 (5.20%)	0.81
Edema	334 (2.90%)	50 (3.10%)	0.66
Oligo-anuria	226 (1.90%)	24 (1.50%)	0.19
**Infective focus**			
Pulmonary site	3873 (33.30%)	545 (33.40%)	0.94
Abdominal site	2780 (23.90%)	383 (23.50%)	0.70
Renal site	1743 (15.00%)	214 (13.10%)	0.05

Variable	No death	Death	p value
Cutaneous site	379 (3.30%)	53 (3.20%)	0.98
Other systemic site	2211 (19.00%)	325 (19.90%)	0.38
**Number of infectious sites**			
0	2606 (22.40%)	365 (22.40%)	0.55
1	7280 (62.60%)	1035 (63.50%)	
2	1547 (13.30%)	213 (13.10%)	
3	180 (1.50%)	17 (1.00%)	
4	13 (0.10%)	1 (0.10%)	
Unconfirmed infective focus	2606 (22.40%)	365 (22.40%)	0.97
**Comorbidities**			
Active malignancy	2461 (21.20%)	350 (21.50%)	0.79
Atrial fibrillation	1201 (12.90%)	193 (15.00%)	0.04
History of MI/CAD	997 (8.60%)	182 (11.20%)	0.001
Heart failure	1098 (9.40%)	237 (14.50%)	<0.001
Dementia	729 (6.30%)	232 (14.20%)	<0.001
COPD	800 (6.90%)	197 (12.10%)	<0.001
Cirrhosis	769 (6.60%)	130 (8.00%)	0.04
Diabetes	1701 (14.60%)	306 (18.80%)	<0.001
Renal insufficiency	1494 (12.90%)	400 (24.50%)	<0.001
Dialysis	284 (2.40%)	27 (1.70%)	0.05
**Outcomes**			
ICU admission	1598 (13.70%)	232 (14.20%)	0.60
